# Monitoring Viral Entry in Real-Time Using a Luciferase Recombinant Vesicular Stomatitis Virus Producing SARS-CoV-2, EBOV, LASV, CHIKV, and VSV Glycoproteins

**DOI:** 10.3390/v12121457

**Published:** 2020-12-17

**Authors:** Maria Fernanda Lay Mendoza, Marissa Danielle Acciani, Courtney Nina Levit, Christopher Santa Maria, Melinda Ann Brindley

**Affiliations:** 1 Department of Infectious Diseases, College of Veterinary Medicine, University of Georgia, Athens, GA 30602, USA; mfl71444@uga.edu (M.F.L.M.); marissa.acciani@uga.edu (M.D.A.); courtneylevit17@uga.edu (C.N.L.); cpsm1@uga.edu (C.S.M.); 2 Department of Population Health, College of Veterinary Medicine, University of Georgia, Athens, GA 30602, USA

**Keywords:** entry, kinetics, luciferase, real-time, live assay, vesicular stomatitis virus, ebola, lassa, chikungunya, coronavirus

## Abstract

Viral entry is the first stage in the virus replication cycle and, for enveloped viruses, is mediated by virally encoded glycoproteins. Viral glycoproteins have different receptor affinities and triggering mechanisms. We employed vesicular stomatitis virus (VSV), a BSL-2 enveloped virus that can incorporate non-native glycoproteins, to examine the entry efficiencies of diverse viral glycoproteins. To compare the glycoprotein-mediated entry efficiencies of VSV glycoprotein (G), Severe acute respiratory syndrome coronavirus 2 (SARS-CoV-2) spike (S), Ebola (EBOV) glycoprotein (GP), Lassa (LASV) GP, and Chikungunya (CHIKV) envelope (E) protein, we produced recombinant VSV (rVSV) viruses that produce the five glycoproteins. The rVSV virions encoded a nano luciferase (NLucP) reporter gene fused to a destabilization domain (PEST), which we used in combination with the live-cell substrate EndurazineTM to monitor viral entry kinetics in real time. Our data indicate that rVSV particles with glycoproteins that require more post-internalization priming typically demonstrate delayed entry in comparison to VSV G. In addition to determining the time required for each virus to complete entry, we also used our system to evaluate viral cell surface receptor preferences, monitor fusion, and elucidate endocytosis mechanisms. This system can be rapidly employed to examine diverse viral glycoproteins and their entry requirements.

## 1. Introduction

Enveloped viruses are covered in a lipid membrane acquired by budding from infected cells. In order for enveloped viruses to infect a cell, the viral membrane must fuse with the cellular membrane, creating a pore through which the viral genome enters the cell cytoplasm. To accomplish fusion, viruses produce fusion proteins studded in their membrane. To date, there are three defined classes of viral fusion proteins, termed class I, II and III [1,2]. While all proteins from all three classes are capable of forming fusion pores between the viral and cellular membranes, they have different properties and requirements [1,2,3]. Here, we produced a panel of recombinant vesicular stomatitis viruses (rVSV) containing five different viral fusion proteins, including representatives from each class. Experiments were designed to monitor entry kinetics and compare the efficiencies of the Lassa (LASV), Ebola (EBOV), severe acute respiratory syndrome coronavirus 2 (SARS-CoV-2), Chikungunya (CHIKV), and VSV glycoproteins.

Class I fusion proteins are translated as single polypeptides that fold into trimeric complexes. A cleavage event produces two subunits, which liberates the fusion peptide and converts the protein into a fusion-ready state. While the pre-fusion structures and fusion triggers for class I proteins are variable, they all adopt a similar post-fusion six-helix bundle (6HB) conformation [1,2]. Coronavirus Spike (S) [4], LASV glycoprotein (GP), and EBOV GP are class I fusion proteins.

**Figure 1:**
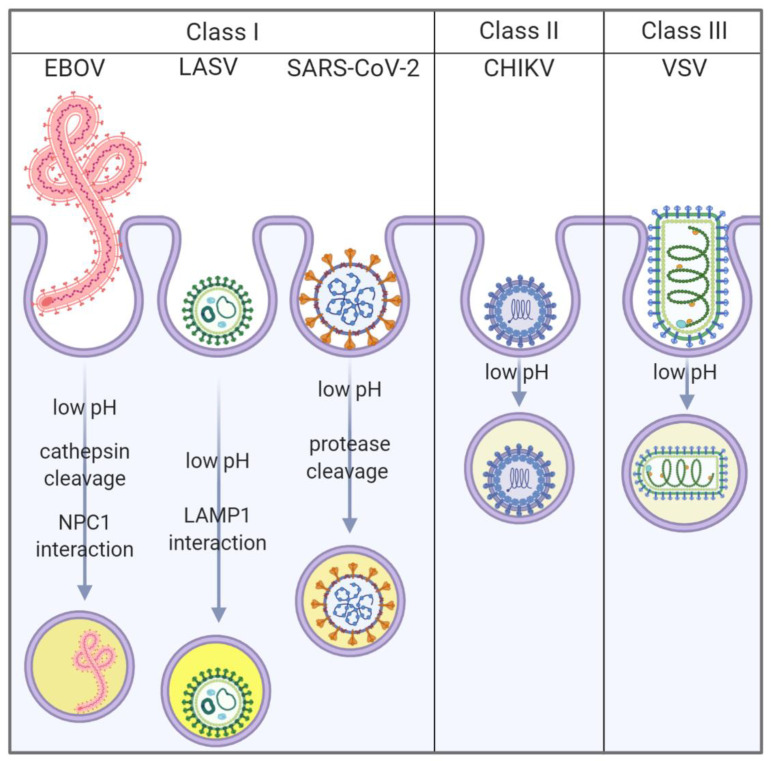
Schematic illustrating the entry pathways for viruses containing class I, class II, or class III fusion proteins, emphasizing the variety of conditions needed to prime the fusion proteins. Chikungunya (CHIKV) and vesicular stomatitis virus (VSV) fusion occurs in early/less acidic endosomes (light yellow, close to the plasma membrane), while Ebola (EBOV), Lassa (LASV), and SARS-CoV-2 fusion occurs in late endosomes/endolysosomes (darker yellow, deeper in the cytoplasm). Created with BioRender.com.

SARS-CoV-2 S initiates infection by interacting with cellular angiotensin-converting enzyme 2 (ACE2) [5,6]. Cellular proteases are required to expose the fusion peptide [7]. In cells with surface proteases, fusion can occur at the plasma membrane in a pH-independent manner [8]. Cells that lack surface proteases mediate entry through low pH activated cathepsins that cleave S in endosomes rather than the cell surface (Figure 1) [9,10]. S cleavage liberates the fusion peptide and triggers conformational changes that enable S to mediate membrane fusion, initiating infection.

Entry of LASV into cells primarily occurs through interaction with α-dystroglycan (αDG) on the cell surface [11,12]. Several attachment factors including heparan sulfate, C-type lectins, TIM-1, and Tyro3, Axl and Mer (TAM) receptor family kinases [13,14,15,16,17] can mediate entry in the absence of functional αDG. Once the virion is within the endolysosomal compartment, low pH and Lysosomal Associated Membrane Protein 1 (LAMP1) induce a conformational change in the glycoprotein [15] resulting in fusion with the endosomal membrane [18]. While many fusion proteins are triggered by low pH (pH < 6), optimal LASV GP fusion activity requires highly acidic conditions (pH 4.5) (Figure 1) [19,20].

While there are no definitive cell surface receptors for EBOV, several types of cell surface proteins including lectins and phosphatidylserine (PtdSer) receptors have been described as important attachment factors to mediate internalization [21,22,23,24]. Once EBOV is in the endolysosomal compartment, a low pH activates cellular proteases that cleave GP [25], facilitating GP interaction with the endosomal receptor Niemann-Pick 1 (NPC1) which induces fusion with the endosomal membrane (Figure 1) [26].

Class II fusion proteins form dimeric pre-fusion complexes rich in β-sheets sitting parallel to the viral membrane [27]. Similar to class I proteins, class II fusion proteins form hair-pin structures during fusion which bring the membranes in close proximity [27]. The CHIKV E2/1 complex protein is a class II fusion protein; E2 is thought to be responsible for receptor binding, while E1 is essential for membrane fusion triggered by low pH in early endosomes (Figure 1) [28]. No receptors have been identified for CHIKV entry into all cells, but several attachment factors can facilitate virion internalization, including Mxra8 [29,30,31,32,33].

Class III fusion proteins form trimers in both the pre- and post-fusion (hairpin-like) conformations [34,35]. Rhabdovirus G proteins, herpesvirus gB [36], and baculovirus gp64 [37] are classified as class III fusion proteins. Unlike the class I and II fusion proteins that are metastable and irreversibly triggered, G folding is reversible and its conformation changes between pre- and post-fusion depending on the pH of the environment [38]. VSV is a prototypical rhabdovirus and its entry mechanism has been studied for decades [39,40]. VSV G mediates entry into an impressive number of cells through interaction with the low-density lipoprotein (LDL) receptor family [41,42], after which low pH in early endosomes triggers membrane fusion (Figure 1) [43].

The entry mechanisms of LASV, EBOV, and SARS-CoV-2 are relatively complex in comparison to those of CHIKV and VSV. SARS-CoV-2 and EBOV fusion requires proteolytic glycoprotein processing, LASV and EBOV fusion requires endosomal receptor interactions, whereas CHIKV and VSV simply require exposure to low pH. To compare glycoprotein-mediated entry efficiencies, we infected a variety of commonly cultured cell lines with recombinant VSV (rVSV) expressing five different glycoproteins: native G, SARS-CoV-2 S, EBOV GP, LASV GP, or CHIKV E. While authentic EBOV, LASV, SARS-CoV-2, CHIKV, and VSV particles have different morphologies, all five glycoproteins are incorporated onto VSV. Our rVSV virions encoded a nano luciferase-PEST (NLucP) reporter gene, which we used in combination with live-cell substrate Endurazine^TM^ to monitor viral entry kinetics in real time. Our data demonstrate both VSV G and CHIKV E can mediate rapid virus entry which is closely followed by SARS-CoV-2 S. Both LASV GP and EBOV GP entry were slower, but EBOV GP-mediated entry occurred more rapidly if the GP was pre-treated with proteases. This system can be rapidly employed to examine diverse viral glycoproteins and their entry requirements.

## 2. Materials and Methods

### 2.1. Cell Lines and Transfections

Vero (vervet kidney cells) constitutively expressing human SLAM/CDw150 (signaling lymphocytic activation molecule 1) (referred to as VeroS) [44] and Baby Hamster Kidney cells (BHK21) stably expressing T7 RNA polymerase [45] were maintained in high-glucose Dulbecco’s Modified Eagle Medium (DMEM; Mediatech, Manassas, VA, USA) supplemented with 5% fetal bovine serum (FBS; Seradigm-VWR, Radnor, PA, USA) (vol/vol). VeroS cells have a significantly higher transfection efficiency compared to VeroE6 and therefore are our preferred VeroS cell line for experiments. Human Embryonic Kidney cells (HEK293T) that express the SV40 large T antigen (kindly provided by Dr. Biao He from University of Georgia) were maintained in high glucose Dulbecco’s Modified Eagle Medium (DMEM) supplemented with 10% FBS (vol/vol). Human haploid cells (HAP1) and HAP1 cells knocked-out for alpha dystroglycan (HΔDAG1) (Horizon Discovery, Cambridge, UK) were maintained in Iscove’s media (Mediatech, Manassas, VA, USA) supplemented with 8% FBS [46]. All cells were kept at 37 °C with 5% CO_2_. BHK-T7 cell transfections were performed with GeneJuice (Millipore, Burlington, MA) and HEK293T cells were transfected with jetPRIME^®^ (PolyPlus, New York, NY, USA) according to the manufacturer’s instructions.

### 2.2. Cloning and Rescue of Viruses

GFP from pVSV∆G/EBOV-GP-GFP and pVSV∆G/VSV-G-GFP molecular clones [47] was replaced with NlucP (Promega^TM^, Madison, WI), utilizing NheI and AvrII restriction sites. To produce pVSV/LASV-GP-Nluc, the codon-optimized protein-coding region of Lassa Josiah strain [46] was amplified with additional MluI and NheI sites which were used to clone into the pVSV∆G_nLucP molecular clone. Similarly, Chikungunya (CHIKV) E protein (strain S27) [48] was amplified with additional MluI and NheI sites to produce pVSV/CHIKV-E-Nluc. pVSV/SARS-CoV-2-S was cloned by adding MluI and NheI sites to the codon-optimized protein-coding region of SARS-CoV-2 S from the Wuhan strain. The S contains an additional 9 residues in the signal peptide since it has been suggested that the starting methionine is upstream of the proposed one [49] and the last 21 residues of the cytoplasmic tail were removed to enhance glycoprotein incorporation into VSV virions [50,51,52]. In addition, D614G was introduced. Rescue of rVSV viruses was completed as previously described [47]. For experiments, the initial recovered virus was passaged onto a T75 of VeroS cells (P2 stocks) and, in some instances, the P2 stock was used to generate more virus (P3 stocks). Stocks and samples were titrated by serial diluting samples in media and determining the median tissue culture infectious dose (TCID_50_) using the Spearman–Karber TCID_50_ method [53]. For some experiments, stocks were titrated with plaque assay on VeroS cells, as previously described [54].

CHIKV-Nluc was made by engineering the Nluc into the CHIKV-181/c25 genome as an additional transcription unit. Nluc was cloned in pSinRep5-181/25ic, a gift from [49] Terence Dermody (Addgene plasmid #60078), using overlapping PCR. To generate viral genomic RNA, the plasmid was linearized with NotI (NEB), in vitro transcribed and capped with the mMESSAGE mMACHINE SP6 Transcription Kit (Invitrogen). Viral RNA (1 µg) was transfected into VeroS cells with Lipofectamine 3000 (Invitrogen) and virus-containing supernatants were collected when cells showed signs of cytopathic effects, approximately 48 h following transfection. For experiments, the initial recovered virus was passaged onto a T75 of VeroS cells (P2 stocks).

### 2.3. Replication Curves of rVSV

VeroS cells were seeded in a 12-well plate at a density of 2 × 10^5^ cells/well. Cells were infected for 1 h at 37 °C with the indicated viruses at an MOI of 0.01 plaque forming unit (PFU)/cell. Media were replaced and supernatants collected at time 0 (immediately) and the indicated time points, stored at −80 °C, and titrated by serial diluting samples in media and determining the median tissue culture infectious dose (TCID_50_) using the Spearman–Karber TCID_50_ method [53].

### 2.4. Entry Kinetics of rVSV into Vero Cells

VeroS cells were seeded at a density of 2 × 10^4^ cells/well in a black-wall clear-bottom 96-well plate. Forty-eight hours post-seeding, Endurazine^TM^ (Promega^TM^, Madison, WI, USA) was diluted 1:100 with DMEM and incubated with the cells for 1 h at 37 °C. Cells were infected at multiple MOIs (25, 5, 1, 0.2, 0.04) PFU/cell with virus in the presence of the substrate for an additional hour. In some experiments, cells were infected with the different viruses at an MOI of 1 and ammonium chloride (NH_4_Cl; 30 mM, pH 7.0) was added at various time points post infection to block subsequent fusion events. After the hour of infection, media were replaced with high-glucose phenol red-free DMEM (supplemented with 5% FBS and 25 mM HEPES). The plate was moved into a pre-warmed (37 °C) plate reader, and luminescence was measured every 10 min at 37 °C with a Glomax^®^ Explorer (Promega^TM^, Madison, WI, USA).

### 2.5. rVSV∆G/LASVGP Entry Kinetics into HAP1 and HΔDAG1 Cells

The day before infection, a 96-well black-wall clear-bottom plate was seeded with 1.5 × 10^4^ cells/well. Entry inhibitors were pre-incubated with cells for 30 min at 37 °C; then Endurazine (Promega, Madison, WI, USA) was added to the cells at the time of infection. HAP1 cells were infected at an MOI of 1 and HΔDAG1 cells were infected with 100 times more virus to reach similar infection rates. Luminescence was measured as described above. Entry inhibitors: 5-(N-Ethyl-N-isopropyl)amiloride (EIPA) (50 µM), dynasore (6.25 µM), nystatin (30 µg/mL), chlorpromazine hydrochloride (0.625 µg/mL) (all from Millipore Sigma, Burlington, MA, USA), dissolved in either dimethyl sulfoxide (DMSO) or water.

### 2.6. Entry Kinetics into HEK293T Expressing Attachment Factors

HEK293T cells were seeded at a density of 3 × 10^4^ cells/well in a 96-well plate. Twenty-four hours post-seeding, cells were transfected with either empty vector, pCS6-Axl (TransOMIC, Huntsville, AL [BC032229]), pCS6-Tyro3 (TransOMIC, Huntsville, AL [BC051756]), TIM-1-GFP [22], pCS6-L-SIGN (TransOMIC, Huntsville, AL, [BC038851]), or pcDNA-hACE2 (hACE2 was a gift from Hyeryun Choe (Addgene plasmid # 1786)) [55]. Two hours post-transfection, half of the media were replaced. Twenty-four hours post-transfection, cells were infected at an MOI of 25 TCID_50_ unit/cell for rVSV∆G/EBOV experiments and MOI 1 for rVSV∆G/SARS-CoV2 experiments in the presence of Endurazine^TM^. Luminescence was measured every 10 min at 37 °C in the presence of HEPES (25 mM).

### 2.7. Thermolysin Cleavage of Virus

rVSV∆G/VSV or rVSV∆G/EBOV were treated as described by the White lab [56]. Virus was incubated with thermolysin (Sigma P1512; 0.1 mg/mL in cleavage buffer (20 mM HEPES, pH 7.5, 20 mM morpholinepropanesulfonic acid, 130 mM NaCl) containing 2 mM CaCl_2_ at 37 °C for 1 h. The reaction was stopped with the addition of ethylenediaminetetraacetic acid (EDTA) (10 mM). Viral particles were then purified from the thermolysin by loading onto an Amicon Ultra spin concentrator (300-kDa cutoff; Millipore) and washing the samples with 5 column volumes of cleavage buffer. Mock-treated samples were processed in the same manner without adding thermolysin. Samples were titrated and no significant differences in infectivity was noted between mock and thermolysin cleaved samples. Cleavage was monitored by sodium dodecyl sulfate–polyacrylamide gel electrophoresis (SDS–PAGE) followed by immunoblot analysis of the viral glycoprotein.

### 2.8. Immunoblots

To detect attachment factors transfected into HEK293T cells, cells were pelleted (800× *g*, 5 min), resuspended in 100 µL of 1X PBS, lysed with 100 µL M2 lysis buffer (50 mM Tris, pH 7.4, 150 mM NaCl, 1 mM EDTA, 1% Triton X-100), and cleared of insoluble material (17,000× *g*, 30 min, 4 °C). Samples were denatured in SDS–UREA buffer (200 mM Tris, pH 6.8, 8 M urea, 5% sodium dodecyl sulfate (SDS), 0.1 mM EDTA, 0.03% bromophenol blue, 1.5% dithiothreitol) for 30 min at 56 °C, separated on a 4–20% Tris-Glycine SDS–PAGE gel (Invitrogen, Waltham, MA) and transferred to PVDF (Polyvinylidene difluoride) membranes. Membranes were incubated with antibodies against GAPDH (Santa Cruz Biotechnology, Dallas, TX [SC-47724, 1:2000]), Axl (R&D Systems Minneapolis, MN, [AF154, 1:2000]), GFP (Thermo Fisher, Waltham, MA, [A-6455, 1:1000]), Tyro3 (R&D Systems Minneapolis, MN, [AF859, 1:1000)], and L-SIGN (Thermo Fisher, Waltham, MA, USA [MA5-21012, 1:200]). Corresponding secondary antibodies conjugated with HRP were used to detect the proteins. Protein signals were detected with West Dura (ThermoFisher, Waltham, MA, USA) and imaged on a BioRad ChemiDocXRS (Bio-Rad, Hercules, CA, USA).

To detect viral envelope in thermolysin-treated and non-treated viral stocks, membranes were incubated with antibodies against EBOV GP (IBT Bioservices, Rockville, MD, USA [0301–001]), VSV G (KeraFast, Boston, MA, USA [EB0010]), and VSV M (KeraFast, Boston, MA, USA [EB0011]).

## 3. Results

### 3.1. Recovery and Replication Rates of the rVSV Viruses

**Figure 2:**
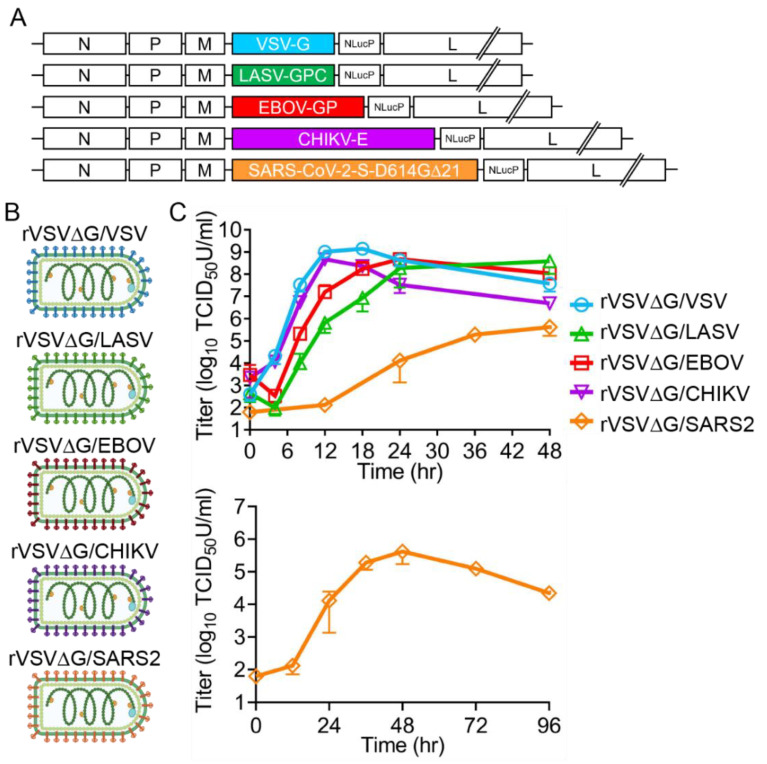
rVSV∆G chimeric viruses′ replication curves. (**A**) Schematic representation of EBOV, LASV, SARS-CoV-2, CHIKV, and VSV envelope and NlucP reporter gene cloned into the molecular clone of VSV. (**B**) Viruses produced from the VSV molecular clones expressing different glycoproteins. Created with BioRender.com (**C**) Multi-cycle replication curves of the viruses on VeroS (MOI 0.01). Lower panel is the complete time course for rVSV∆G/SARS. Each experiment was repeated three independent times. Data shown are the averages and SEM.

To compare the entry efficiencies of EBOV, LASV, SARS2, CHIKV, and VSV, we cloned the glycoproteins into the molecular clone of VSV and inserted an additional transcriptional unit encoding a reporter gene, NlucP, in a post-envelope location (Figure 2A). Viruses were recovered (Figure 2B) and all subsequent experiments were completed with the stocks produced from the second or third passage amplified on VeroS cells. To monitor virus production over time, we performed multi-cycle replication curves, infecting cells at an MOI of 0.01 (Figure 2C). VSV G and CHIKV E produced the highest titers of virus, and peak titers were observed sooner than the other rVSV viruses at 12 h following infection. EBOV GP reached peak titers 24 h following infection, and LASV required 48 h. rVSV∆G/SARS2 replication was severely reduced compared to the other glycoproteins. While rVSV∆G/SARS2 titers peaked at 48 h following infection, the peak titer was orders of magnitude lower than peaks found with the other glycoproteins (Figure 2C).

### 3.2. Kinetics of rVSV∆G/VSV Luciferase Expression

**Figure 3:**
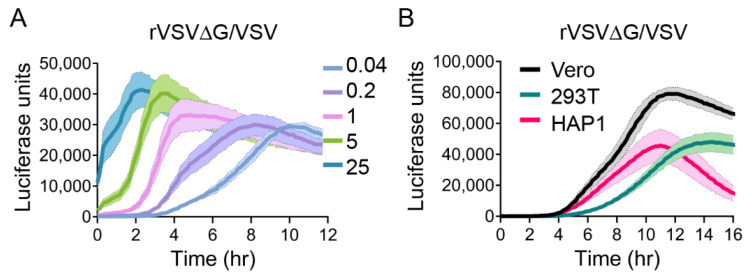
rVSV∆G/VSV entry kinetics. (**A**) VeroS cells were infected with rVSV∆G/VSV at multiple MOIs, inoculum was removed after 1 h, and monitored for luciferase production over time. (**B**) VeroS, HAP1, and HEK293T cells were infected with rVSV∆G/VSV (MOI 1), inoculum was not removed, and monitored for luciferase production over time. Each experiment was repeated in duplicate, three independent times. Data shown are the averages of averages and corresponding SEM.

To examine how quickly we can detect viral entry in our rVSV system, we used the live-cell luciferase substrate Endurazine™, which requires cellular esterase cleavage in order to react with the rVSV-encoded NLucP reporter gene and produce luminescence. We measured luminescence over a shorter time course than that of our replication curves in order to capture the first 1–2 rounds of replication. We first observed rVSV containing its native glycoprotein (G). VSV G is known to efficiently and quickly enter cells and fuse out of early endosomes [57]. We first assessed how changes in MOI values could impact the kinetics of luciferase expression of rVSV∆G/VSV, by infecting VeroS cells at an MOI of 25, 5, 1, 0.2, and 0.04. The virus was added to the cells for one hour, then both the inoculum and luciferase substrate were removed, and the cells were placed in the pre-warmed plate reader; therefore, only particles that bound within the hour could initiate infection. At 1 h post-infection, luciferase production from cells infected at the highest MOI (25) already displayed a signal value of ~10,000 units and quickly peaked 3 h post-infection (Figure 3A). At an MOI of 5, the luciferase signal quickly increased and peaked 3.5 h post-infection. When adding fewer particles to the cells, we could detect a clear eclipse phase; with approximately 1 virus per cell (MOI 1), a luciferase signal was detected about two hours after removal of the inoculum, and the signal rapidly rose and displayed a maximum peak at 5 h post infection. This suggests the time from infection to peak protein production occurs within 5 h of infection, which is consistent with previous studies of VSV replication kinetics [57]. At lower MOI infections, we observed an almost steady increase in luciferase production, which peaked late, between 8 and 10 h. We attribute this to virus spreading to uninfected cells, eventually exhausting the luciferase substrate.

To compare luciferase activity across different cell types, we seeded VeroS, HAP1, and HEK293T cells at the same cell density and infected them with rVSV∆G/VSV (MOI 1) (Figure 3B). Because HAP1 and HEK293T cells did not withstand washing, the viral inoculum was not removed in this experiment. When we compare the VeroS MOI 1 data between Figure 3A,B, we can see how differently the luciferase signal accumulates when the inoculum is removed versus left on the cells. The data suggest that additional virus continues to bind and enter cells throughout the course of the experiment, enabling more luciferase to be produced. In addition, the luciferase substrate was also present for the length of the infection in Figure 3B, which was attributed to the higher peak values that were produced later in the infection. Within this experiment, VeroS and HAP1 cells displayed similar initial signals and peak times (between 11 and 12 h post-infection); however, VeroS cells reached a higher peak than HAP1 cells. Luciferase production in HEK293T was slower than in VeroS and HAP1 cells; it reached a similar peak value to the HAP1 cell line, but about 3 h later (Figure 3B).

### 3.3. Kinetics of rVSV∆G/LASV Luciferase Expression

**Figure 4:**
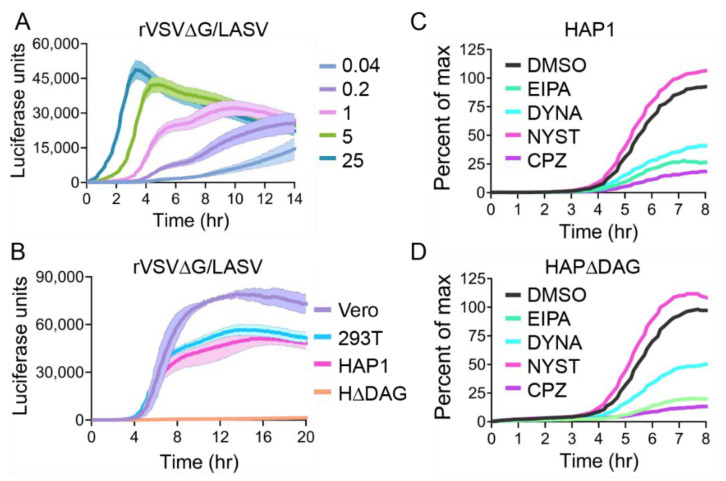
rVSV∆G/LASV entry kinetics: (**A**) VeroS cells were infected with rVSV∆G/LASV at multiple MOIs, inoculum was removed after 1 hr, and monitored for luciferase production over time. (**B**) VeroS, HEK293T, HAP1, and H∆DAG cells were infected with rVSV∆G/LASV (MOI 1), inoculum was not removed, and monitored for luciferase production over time. (**C**) HAP1 cells and (**D**) H∆DAG cells were infected with rVSV∆G/LASV (H∆DAG cells required 100 times as much virus) in the presence of commonly used entry inhibitors. Results are displayed as percentage of the maximum signal seen with mock treatment. Each experiment was repeated in duplicate, three independent times. Data shown are the averages of averages and corresponding SEM.

Once we established how quickly we could detect a luciferase signal when rVSV was entering using the VSV G, we compared how quickly rVSV entry occurs when the LASV GP is mediating entry. Unlike VSV G, which fuses with early endosomal membranes, LASV fusion requires low pH values found in late endosomes/lysosomes and therefore we predicted that luciferase production would both start and peak at later time points than VSV G. To determine signal peak and relative strength, we infected VeroS cells at an MOI of 25, 5, 1, 0.2, and 0.04. While VSV G-mediated entry at high MOI (25) peaked at two hours, LASV GP was slower and displayed a maximum signal peak at 3.5 h (Figure 4A). At lower MOIs, we detected a slight plateau in the signal accumulation between 5 and 7 h, after which a second round of infection induced more luciferase production (Figure 4A).

LASV GP interacts with αDG to efficiently enter cells, but can also enter cells through additional attachment factors. We compared LASV entry into 293T and HAP1 cells, which both produce αDG, as well as VeroS cells, which lack properly glycosylated αDG [12,16], and HAP1 cells knocked out for αDG gene (H∆DAG) (Figure 4B). Both HAP1 and HEK293T cell lines that contain αDG displayed overlapping kinetics of luciferase expression over the course of the experiment (Figure 4B). VeroS cells contain high levels of PtdSer receptors that facilitate LASV entry in the absence of αDG [16], and the rate of signal production closely followed the αDG producing cells, but produced higher overall luciferase levels. The H∆DAG did not produce significant signals above the background for the first 12 h, but the signal eventually separated from the background, although it remained low.

Previous studies have demonstrated that rVSV∆G/LASV can enter HΔDAG cells if enough virus is added to the culture [15,46]. Despite the lack of αDG, we could detect similar levels of luciferase production in the HΔDAG1 by increasing the viral load by 100-fold over the level added to HAP1 cells (Figure 4C,D). To determine if virion internalization occurs through similar or distinct mechanisms if particles interact with αDG or other attachment factors, we infected HAP1 and HΔDAG1 at an MOI of 1 and 100, respectively, and measured luminescence in the presence of chlorpromazine, EIPA, dynasore, or nystatin entry inhibitors (Figure 4C,D). After normalization to control (DMSO), both HAP1 and HΔDAG1 cells displayed similar patterns of inhibition. Viral entry into both cell types is strongly inhibited by macropinocytosis (EIPA) and clathrin-mediated (CPZ) endocytosis inhibitors, but is not affected by nystatin (NYST) which blocks caveolin-mediated internalization.

### 3.4. Kinetics of rVSV∆G/EBOV Luciferase Expression

**Figure 5:**
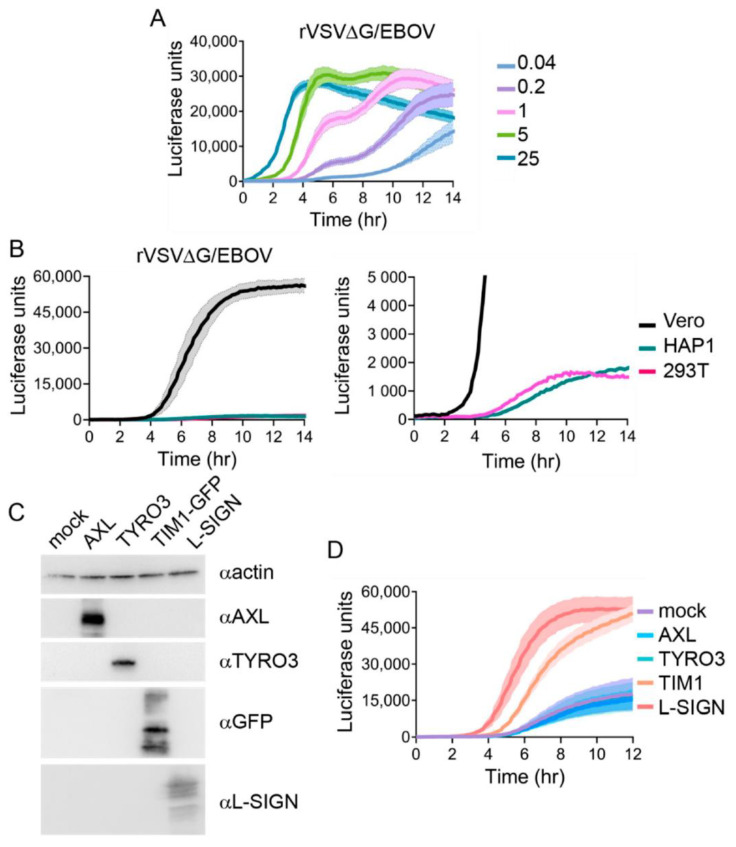
rVSV∆G/EBOV entry kinetics: (**A**) VeroS cells were infected with rVSV∆G/EBOV at multiple MOIs, inoculum was removed after 1 h, and monitored for luciferase production over time. (**B**) VeroS, HEK293T, and HAP1 cells were infected with rVSV∆G/EBOV (MOI 1), inoculum was not removed, and monitored for luciferase production over time. Right panel zooms in to display the signals observed in HAP1 and HEK293T cells. (**C**) Immunoblots demonstrating production of the transfected receptors in HEK293T cells. (**D**) HEK293T cells transfected with the indicated receptors were infected with rVSV∆G/EBOV and monitored for luciferase production over time. Each experiment was repeated in duplicate, three independent times. Data shown are the averages of averages and corresponding SEM of three independent trials.

Next, we examined rVSV∆G/EBOV entry. Like LASV, EBOV entry requires virions to traffic into more mature endosomes before fusion can occur. EBOV must first undergo proteolytic cleavage by endosomal cathepsins before it can interact with the endosomal receptor NPC1 [25,26]. Infection of VeroS cells with rVSV∆G/EBOV at an MOI of 25 (PFU/cell), resulted in a signal peak at 4.5 h post-infection and, at an MOI of one, the signal peaked around 7 h (Figure 5A), and was therefore slower than both VSV G and LASV GP-mediated entry.

rVSV∆G/EBOV infection of HAP1 and HEK293T yielded comparably low luciferase activity to VeroS cells (Figure 5B). While the signals were less than 4% of that observed in VeroS cells, the signal peaks were reached between 10 and 12 h following infection. Previous reports suggest HEK293Ts are not highly permissive for EBOV entry [22], yet the NPC1 receptor was identified by infecting HAP1 cells with rVSV∆G/EBOV [26]. Attachment factors play a key role in the facilitation of EBOV entry [22,23,24]. Since HEK293T cells naturally lack PtdSer receptors and C-type lectins, we wanted to evaluate whether the luciferase signal could be enhanced by producing attachment factors. We transfected HEK293Ts with plasmids encoding L-SIGN, Axl, Tyro3, and TIM-1-GFP and confirmed protein production with immunoblots (Figure 5C). Our results indicate that HEK293T cells transfected with L-SIGN and TIM-1-GFP reached a higher signal value upon rVSV∆G/EBOV infection in comparison to control group, with signals peaking first when L-SIGN was present (Figure 5D). Surprisingly, HEK293T cells transfected with Axl and Tyro3 remained at baseline level and no significant change was observed. Both Tyro3 and Axl are TAM family members that require adaptor proteins such as Gas6 to bind to PtdSer and facilitate entry. Gas6 is found in fetal calf serum (FCS) [58]. While the experiment contained FCS, there may have been insufficient levels to link virus to Tyro3 or Axl and facilitate entry.

### 3.5. Kinetics of rVSV∆G/SARS2 Luciferase Expression

**Figure 6:**
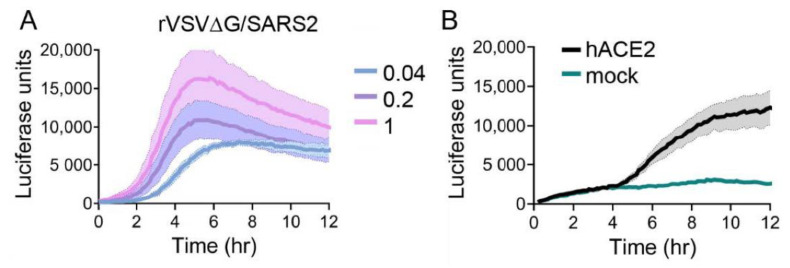
rVSV∆G/SARS2 entry kinetics: (**A**) VeroS cells were infected with rVSV∆G/SARS2 at multiple MOIs, inoculum was removed after 1 hr, and monitored for luciferase production over time. (**B**) HEK293T were either mock transfected or transfected with ACE2 and infected with rVSV∆G/SARS2 and luciferase production was monitored over time. Each experiment was repeated in duplicate, three independent times. Data shown are the averages of averages and corresponding SEM of three independent trials.

rVSV∆G/SARS2 did not replicate to the high titers seen with the other glycoproteins and therefore we were unable to achieve high-MOI infections. Titers permitted infections at an MOI of 1, and signal accumulated relatively fast, peaking at 5 h post infection (Figure 6A) At lower MOIs, we did not observe a second wave of luciferase produced, as seen with EBOV and LASV (Figure 4A and Figure 5A), which may relate to the poor titers produced with rVSV∆G/SARS2 (Figure 2C).

SARS2 S protein interacts with ACE2 to facilitate entry into cells [7]. By transiently producing ACE2 in 293T cells that naturally lack ACE2 expression, we can detect a significant luciferase signal compared to background levels starting at 5 h following infection (Figure 6B). rVSV∆G/SARS2 particles did display higher background signals than the other rVSV. We attribute this to the low titers, which required higher volumes of inoculum to be added to the experiment.

### 3.6. Kinetics of rVSV∆G/CHIKV and CHIKV Luciferase Expression

**Figure 7:**
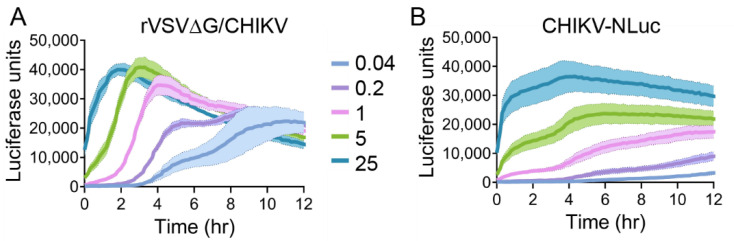
rVSV∆G/CHIK and CHIKV entry kinetics. VeroS cells were infected with rVSV∆G/CHIKV (**A**) or CHIKV (**B**) at multiple MOIs, inoculum was removed after 1 h and monitored for luciferase production over time. Each experiment was repeated in duplicate, three independent times. Data shown are the averages of averages and corresponding SEM of three independent trials.

We performed the same multi-MOI experiment on VeroS cells, utilizing rVSV expressing the wild type CHIKV envelope (S27), and compared the rVSV signals to CHIKV (181/c25) that produces nano luciferase from a subgenomic RNA. CHIKV-NLuc encodes for nano-luciferase lacking the PEST domain, whereas the rVSV constructs encode for NLucP, which is rapidly degraded. rVSV∆G/CHIKV luciferase kinetics closely mirrored rVSV∆G/VSV with signals rapidly increasing and peaking at 4 h post-infection at an MOI of 1. The two lowest MOIs (0.2 and 0.04) display a multistep curve, suggesting the first round of replication peaks at about 5 h post-infection followed by a second peak approximately 5 h later (Figure 7A). CHIKV-nLuc curves followed different trends than the rVSV. Altering the amount of virus in the inoculum caused a clear difference in signal (Figure 7B). While the time frame for rVSV genome to accumulate a luciferase signal was a few hours, CHIKV-nLuc signals rapidly produced signal within 1 h of infection. That signal remained relatively stable until 4 h post-infection, when an increase in the signal produced was observed.

### 3.7. Comparative Analysis of rVSV Viruses’ Kinetics of Luciferase Expression

**Figure 8:**
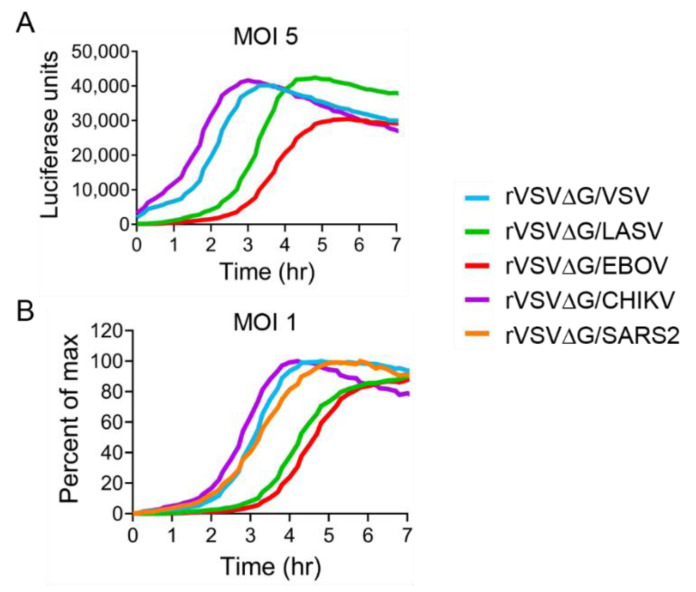
Comparative analysis of rVSV viruses. Data displayed in Figure 3, Figure 4, Figure 5, Figure 6 and Figure 7 were replotted to compare the entry kinetics of the five viruses. MOI 5 data are displayed (**A**). To compare time to peak, the data at a MOI of 1 was replotted as percent of max (**B**).

To compare all the rVSV viruses, we overlayed the luciferase signals produced at high MOIs (5) (Figure 8A). Surprisingly, CHIKV envelope-mediated entry induced slightly faster luciferase signals than even the native VSV glycoprotein. Both LASV- and EBOV-mediated signals peaked more than an hour later. To compare all five glycoproteins, we focused on the time it took to reach peak signal at an MOI of 1 (Figure 8B). EBOV- and LASV-mediated luciferase signals peak later in the infection, whereas VSV, CHIK and SARS2 are able to produce higher luciferase signals earlier in the infection. 

**Figure 9:**
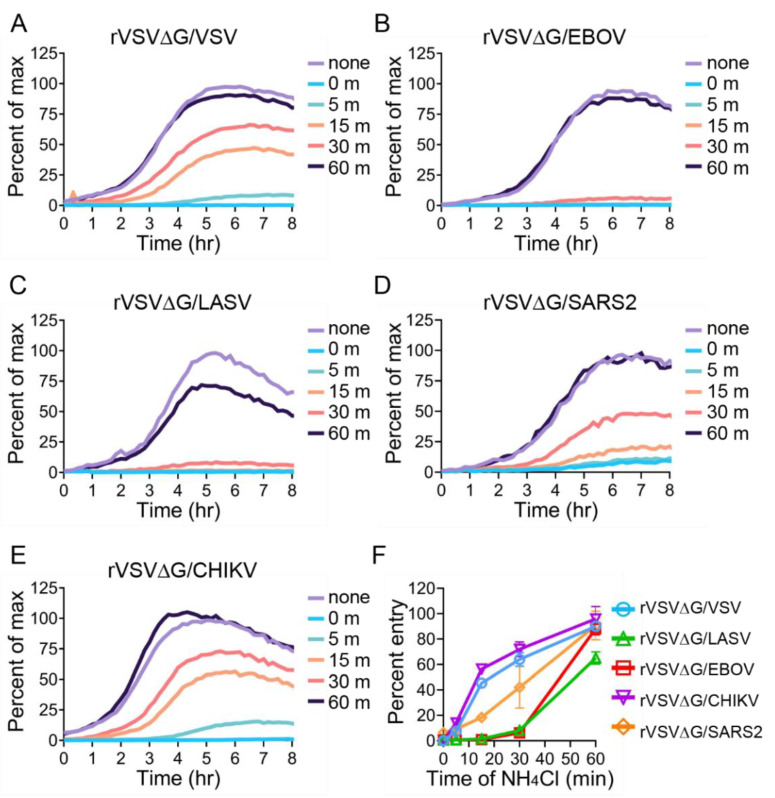
rVSV entry in presence of ammonium chloride. VeroS cells were infected with (**A**) rVSV∆G/VSV, (**B**) rVSV∆G/EBOV, (**C**) rVSV∆G/LASV, (**D**) rVSV∆G/CHIKV, and (**E**) rVSV∆G/SARS2 (MOI 1). At the indicated time points ammonium chloride was added to prevent low pH mediated fusion. One-hour post infection, the inoculum was removed and luciferase production was monitored over time. (**F**) Percent of max luciferase produced 6 h post-infection was plotted to compare how quickly the different glycoproteins escape the ammonium chloride entry block. Each experiment was repeated in duplicate, three independent times. Data shown are the averages of averages and SEM of three independent trials. Note the SEM was not shown in (**A**–**E**) for clarity.

In all of these experiments, luciferase is produced by the viral replication machinery after the internal VSV ribonucleoprotein complex is delivered into the cytoplasm following membrane fusion. Because the five glycoproteins range in size, (e.g., SARS2 S protein is more than twice the size of VSV G), we wanted to confirm that the delay in luciferase production correlated to the virions fusing with the endosomal membrane. All five of the glycoproteins enter VeroS cells through an endosomal route, and fusion is triggered through low-pH-dependent processes [9,20,25,59,60]. Therefore, we infected VeroS cells at an MOI of 1 and added ammonium chloride (NH_4_Cl), a lysosomotropic agent, that quickly prevents endosomal acidification and should prevent luciferase production if the virions have not undergone fusion. Adding NH_4_Cl at the time of infection prevented luciferase production for all viral glycoproteins (Figure 9). Both VSV and CHIKV fusion occurred quickly, with approximately half of the particles escaping the NH_4_Cl block within 15 min (Figure 9A,E). SARS2 required 30 min (Figure 9D) for half of the particles to escape the endosomal compartment. Both EBOV and LASV displayed a much slower entry pathway, with the majority of particles being blocked when adding NH_4_Cl 30 min after infection (Figure 9B,C). The percent of entry observed at 6 h post infection was plotted to compare the five glycoproteins. Similar to the previous experiments, VSV and CHIKV had faster fusion kinetics, followed by SARS2 and, lastly, EBOV and LASV (Figure 9F).

### 3.8. rVSV∆G/EBOV Kinetics of Luciferase Expression Shifts with Thermolysin Treatment

**Figure 10:**
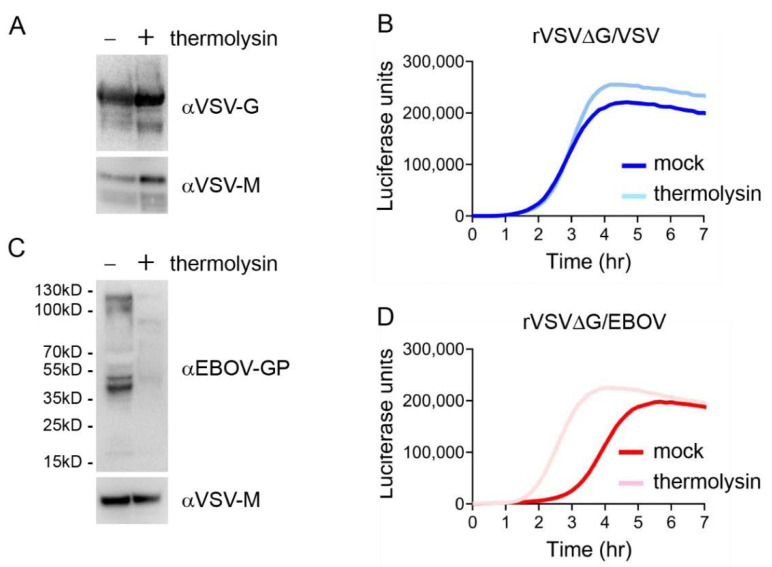
rVSV∆G/EBOV entry occurs faster with proteolytically processing GP. Immunoblots of rVSV∆G/VSV (**A**) or rVSV∆G/EBOV (**C**) treated or mock-treated with thermolysin. VeroS cells were infected with rVSV∆G/VSV (**B**) or rVSV∆G/EBOV (**D**) treated or mock-treated with thermolysin and luciferase production was monitored over time. The experiments were repeated in duplicate, three independent times. Data shown are the averages of technical replicates from one trial and is representative of the experiment.

rVSV∆G/EBOV entry required significantly more time than rVSV∆G/VSV. While rVSV∆G/VSV can be triggered by exposure to low pH, EBOV GP must undergo proteolytic processing in the late endosomes before it can interact with its receptor and trigger fusion [25]. EBOV GP proteolysis can be mimicked by treating the viral particles with thermolysin, a metallopeptidase [61]. To determine if cathepsin cleavage is a rate-limiting factor in the entry kinetics of EBOV GP we treated either rVSV∆G/VSV or rVSV∆G/EBOV particles with thermolysin before infecting cells and monitoring luciferase activity. While thermolysin treatment did not alter VSV G levels on the particle, thermolysin decreased the levels of full-length EBOV GP (Figure 10A,C). Unfortunately, our EBOV GP antibody did not appear to detect the 19kDa GP fragment. Thermolysin treatment did not alter virion titers and both untreated and treated rVSV∆G/VSV particles produced luciferase levels at similar rates (Figure 10B). Thermolysin-treated rVSV∆G/EBOV particles displayed a shift in luciferase production, with treated particles producing luciferase two hours earlier than untreated particles and similarly reaching a signal peak two hours earlier (Figure 10D).

## 4. Discussion

The production of replication competent chimeric VSV particles has been used extensively to examine the virus entry of highly pathogenic viruses in a BLS2 system [62,63,64]. These particles are also being used as vaccine vectors, with the rVSV-ZEBOV becoming the first licensed EBOV vaccine [65,66]. We employed this system to compare the efficiencies of different viral glycoproteins in live cells. All of the viruses contain the same internal VSV replication machinery and only differ in the outer glycoprotein. Therefore, we monitored the production on a NlucP reporter construct as a surrogate for virus entry, assuming that the VSV machinery would have similar transcription kinetics once the viral core was delivered to the cytoplasm. VSV G, CHIKV E, and SARS-CoV-2 S-mediated entry at a faster rate than EBOV and LASV. Although the genome length varied among the viruses (Figure 1), the differences in length did not correlate with changes in luciferase production, suggesting that the additional length did not significantly alter the rate of luciferase production. The CHIKV E protein open reading frame is about twice the length of the VSV G, yet both viruses mediated luciferase production at very similar rates (Figure 3A and Figure 7A). Additionally, the time frame observed monitoring luciferase production closely correlated with the time-of-addition assays that blocked low pH-dependent fusion. VSV G and CHIK E particles were able to escape the low-pH step in viral entry at a faster rate than SARS-CoV-2 S, which was faster than the comparatively slow entry mediated by LASV GP and EBOV GP.

While all five viral glycoproteins require low pH for entry, LASV GP and EBOV GP require endosomal receptor binding and EBOV GP requires proteolytic processing [15,20]. Because we observed that rVSV∆G/EBOV entry was slower than rVSV∆G/LASV entry, we examined if EBOV GP proteolytic processing delays rVSV∆G/EBOV entry. We pre-cleaved rVSV∆G/EBOV particles with thermolysin and found that pre-cleaved particles produced luciferase significantly earlier than non-cleaved particles, suggesting that proteolysis is a rate-limiting step in EBOV entry. SARS-CoV-2 S also requires proteolytic cleavage, either at the plasma membrane or in endosomes. Here, we infected VeroS cells, which lack TMPRSS2, a cell surface protease required for S cleavage at the plasma membrane [7,8]. However, cathepsins found in the endosomal compartments can compensate, enabling entry [9,10]. With both EBOV and SARS-CoV-2 requiring endosomal cathepsin cleavage to initiate fusion in our system, we were surprised that SARS-CoV-2 entry occurred at a faster rate than EBOV. In addition to EBOV GP requiring interaction with NPC1 following cathepsin cleavage, additional, undefined steps are needed to trigger EBOV GP [26,56,67], whereas, for SARS-CoV-2, S proteolysis is the primary trigger of S conformational changes.

SARS-CoV-2 Spike-containing rVSV particles spread poorly in our multi-step replication curves and produced significantly lower titers than the other chimeric viruses. Surprisingly, the first round of entry was comparably efficient and produced luciferase as quickly as rVSV/VSV; however, we did not observe a second wave of luciferase production. This may be due to poor SARS-CoV-2 S incorporation onto particles causing inefficient spread of the virus.

We compared rVSV∆G/VSV, rVSV∆G/EBOV, and rVSV∆G/LASV entry in VeroS, HAP1 and HEK293T cells. With all three viruses, infection in VeroS cells resulted in significantly higher luciferase levels, whereas luciferase production in HEK293T and HAP1 were equally reduced (Figure 3B, Figure 4B and Figure 5B). Both rVSV∆G/VSV and rVSV∆G/LASV entered all three cell types with similar efficiencies, suggesting the higher overall luciferase values in VeroS cells may be attributed to other factors such as increased cell size (each VeroS cell is approximately twice as large as a HAP1 or HEK293T cell) or greater VeroS Endurazine^TM^ processing. The high sensitivity of NlucP-Enduazine^TM^ system enabled the detection of virus replication even when very few particles were efficiently entering (Figure 4B and Figure 5B), suggesting that a slight increase in VeroS Endurazine processing may result in greater amplified luciferase signals.

We also compared rVSV∆G/CHIK and CHIKV entry efficiencies. Cells infected with CHIKV produced luciferase activity more rapidly than rVSV∆G/CHIK and rVSV∆G/VSV, which is likely due to CHIKV’s positive sense RNA genome. The initial spike in luciferase from the first round of CHIKV replication remained relatively stable for three hours, after which a second round of replication was detected by an increase in luciferase activity (Figure 7B). rVSV∆G/CHIK, which contains a non-native glycoprotein and a negative-sense RNA genome, produced luciferase at a slower rate (Figure 7A). Luminescence also continued to increase throughout the first round of rVSV∆G/CHIK infection, rather than remaining relatively stable as seen with CHIKV. One caveat when comparing the data is that the rVSV encoded for NLucP includes a PEST protein degradation domain to reduce background from virus inoculum and ensure the protein is actively being produced during the assay. CHIKV-NLuc does not contain the PEST domain, so the rapid signal with little accumulation over the first three hours may be due to the high activity and low turnover of NLuc in this viral infection. Future comparisons should include the same NLucP reporter in both viruses.

When comparing the entry efficiency at different MOIs, both the virus and the substrate were removed after one hour of infection. If we compare this method to experiments in which the virus and substrate were not removed, we find both the time to signal peak and the height of the signal differ. If we compare viral titers when virus is removed from the system after one hour to experiments where the inoculum is never removed, we observe approximately a 10-fold difference, suggesting additional virions continue to enter. Therefore, when the virus is not removed, there is more asynchronous entry, which may alter the signal accumulation. Additionally, the removal of the substrate may blunt signal peaks if the substrate becomes limiting. It is important to only compare data from experiments with similar conditions. Other groups have designed assays to monitor early stages in viral replication, but because each assay differs (i.e., cell type, reporter, assay sensitivity), comparing this assay with previous experiments is difficult.

## 5. Conclusions

In conclusion, we can use rVSV viruses to compare the entry kinetics of a wide range of viral glycoproteins. This live-cell assay quickly enables one to compare viral entry conditions in various cell lines and is highly sensitive. The method could easily be adapted for screening purposes.
